# The British Columbia Healthy Connections Project: findings on socioeconomic disadvantage in early pregnancy

**DOI:** 10.1186/s12889-019-7479-5

**Published:** 2019-08-22

**Authors:** Nicole L. A. Catherine, Rosemary Lever, Debbie Sheehan, Yufei Zheng, Michael H. Boyle, Lawrence McCandless, Amiram Gafni, Andrea Gonzalez, Susan M. Jack, Lil Tonmyr, Colleen Varcoe, Harriet L. MacMillan, Charlotte Waddell, For the British Columbia Healthy Connections Project Scientific Team

**Affiliations:** 1 Children’s Health Policy Centre, Faculty of Health Sciences, Simon Fraser University, Room 2431, 515 West Hastings Street, Vancouver, BC V6B 5K3 Canada; 2 Offord Centre for Child Studies, Faculty of Health Sciences, McMaster University, Hamilton, Ontario Canada; 3 Faculty of Health Sciences, Simon Fraser University, Burnaby, BC Canada; 4 Faculty of Health Sciences, McMaster University, Hamilton, Ontario Canada; 5 School of Nursing, McMaster University, Hamilton, Ontario Canada; 6 Public Health Agency of Canada, Ottawa, Ontario Canada; 7 School of Nursing, University of BC, Vancouver, BC Canada; 8 Departments of Psychiatry and Behavioural Neurosciences and of Pediatrics, Offord Centre for Child Studies, Faculty of Health Sciences, McMaster University, Hamilton, Ontario Canada

**Keywords:** Pregnancy, Adolescents, Maternal health, Socioeconomic disadvantage, Cumulative disadvantage

## Abstract

**Background:**

Maternal exposure to socioeconomic disadvantage increases the risk of child injuries and subsequent child developmental and mental health problems — particularly for young mothers. To inform early intervention planning, this research therefore aimed to describe the health and social adversities experienced by a cohort of girls and young women in early pregnancy in British Columbia (BC), Canada.

**Methods:**

Participants were recruited for the BC Healthy Connections Project (BCHCP), a randomized controlled trial examining the effectiveness of Nurse-Family Partnership, a home visitation program, in improving child and maternal outcomes. Baseline data were collected from 739 participants on trial entry. Participants were selected on the basis of preparing to parent for the first time and experiencing socioeconomic disadvantage. Analyses involved descriptive statistics and age-group comparisons.

**Results:**

Most participants reported having low income (84%), having limited education (52%) and being single (91%) at trial entry. Beyond these eligibility criteria, other health and social adversities included: housing instability (52%); severe anxiety or depression (47%); other diagnosed mental disorders (22%); prenatal nicotine and cannabis use (27 and 21%); physical health problems (20%); child maltreatment when younger (56%); and intimate partner violence recently (50%). As well, few (29%) had received income assistance entitlements. More than two thirds (70%) were experiencing four or more forms of adversity. Age-group differences were observed for cognitive functioning, being single, low income, limited education, psychological distress and service use (*p*-value ≤0.05).

**Conclusions:**

This cohort was selected on the basis of socioeconomic disadvantage. Yet all participants were experiencing substantial added adversities — at higher rates than other Canadians. Furthermore, despite Canada’s public programs, these pregnant girls and young women were not being adequately reached by social services. Our study adds new data to inform early intervention planning, suggesting that unacceptably high levels of socioeconomic disadvantage exist for some young British Columbians. Therefore greater health and social supports and services are warranted for these young mothers and their children.

**Trial registration:**

Registered August 24, 2012 with ClinicalTrials.gov Identifier: NCT01672060. Active not recruiting.

## Background

Socioeconomic disadvantage poses challenges to the wellbeing of both mothers and children [1–3]. In particular, adolescent mothers (aged 19 years or younger) are more likely to experience interrupted education, lower workforce participation, lower income, unstable housing, and associated physical and mental health and cognitive problems [4, 5]. Children born to adolescent mothers, in turn, are at greater risk for preterm birth, childhood injuries and subsequent developmental and mental health problems [6–8]. Similarly, children born to young mothers (aged 20–24 years) who are experiencing socioeconomic disadvantage (such as having low income, having limited education or having limited social supports) are also at greater risk for injuries and subsequent developmental and mental health problems [2, 9–12]. Other health and social adversities associated with maternal socioeconomic disadvantage in general include depression, prenatal substance use and exposure to intimate partner violence (IPV), which also adversely influence the developing child [11, 13–15].

Yet the socioeconomic disadvantage that underlies many childhood mental and physical health problems is socially produced and therefore may be amenable to intervention [16–18]. Providing children with a better start in life, beginning before or during pregnancy and continuing in the early years, promotes healthy development and results in greater societal benefits compared to later remediation of health and social problems [18–22]. It is therefore crucial to identify opportunities for intervening “upstream” — well before avoidable adversities occur and subsequent health and social problems begin.

One approach is to identify disadvantaged populations in early pregnancy and examine how avoidable adversities may be offset or muted by specific prevention interventions aimed at improving the life course trajectories for both children and mothers [18–22]. Reducing socioeconomic disadvantage and improving parenting — through providing better supports for pregnant girls and young women and new mothers — is a powerful mechanism for supporting healthy development throughout the lifespan [1, 23]. Yet data describing populations of disadvantaged young mothers-to-be in Canada have been limited, in turn, limiting the data available to inform the development and provision of effective maternal and child services that are proportionate to the level of disadvantage or need [18]. As well, disadvantaged groups have often been characterized as “hard-to-reach” rather than “need-to-reach,” further hampering intervention efforts [24]. To inform intervention planning, this research therefore aimed to describe the health and social adversities experienced by a cohort of 739 pregnant girls (14–19 years) and young women (20–24 years) in British Columbia (BC), Canada.

## Methods

### Study design

We report on baseline data from the BC Healthy Connections Project (BCHCP), a randomized controlled trial (RCT) evaluating the effectiveness of the Nurse-Family Partnership (NFP) program compared with existing health and social services [25]. NFP involves nurses providing intensive home visits with young, low-income, first-time mothers, starting in early pregnancy and continuing until children reach age 2 years [23]. Developed in the United States, NFP has been shown to reduce child injuries and improve children’s mental health and development, while also improving mothers’ life circumstances, especially for those experiencing the highest levels of disadvantage [23]. We also compared data across both age groups (14–19 and 20–24 years) to ascertain similarities or differences in experiences of disadvantage in early pregnancy, and to determine whether we in fact had reached those whom NFP is most intended to help.

### Participants

We used baseline (pre-randomization) data for 739 participants enrolled in the BCHCP. Participants were eligible if they: were in early pregnancy (less than 28 weeks gestation); preparing to parent for the first time; were young (24 years or younger); and were experiencing socioeconomic disadvantage, (a risk factor for child injuries, the trial primary outcome indicator). Indicators of disadvantage included: having low-income (receiving income assistance, or experiencing homelessness, or finding it very difficult to live on total household income with respect to food or rent); having limited education (less than high school); or preparing to parent while single (not married or not living common-law for one year or more). Pregnant girls aged 14–19 years were deemed to automatically meet disadvantage criteria due to their young age; young women aged 20–24 years were required to meet two of three indicators. Previous NFP trials in other countries enrolled pregnant girls living with low income [26], or girls and young women (less than 26 years old) experiencing disadvantage [27].

**Fig. 1:**
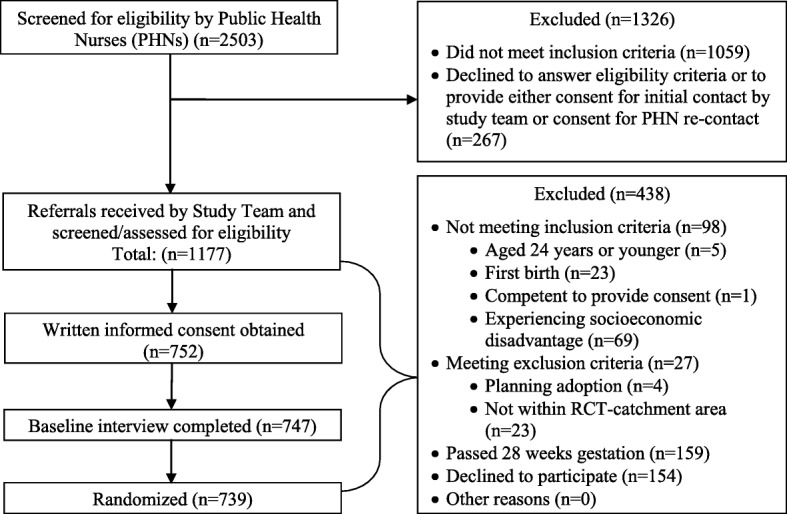
Participant flow

Referrals came from public health units at four regional BC Health Authorities (Fraser, Interior, Island and Vancouver Coastal Health). Recruitment targets were met after three years (that is, 60% were reached by public health nurses, of which 60% were enrolled, which comprised one third of all potentially eligible participants). Baseline data were gathered during in-person research interviews conducted in participants’ homes between October 2013 and December 2016. Detailed trial information is described in the RCT study protocol [25]. Figure 1 shows participant flow. The BCHCP trial adheres to Consolidated Standards of Reporting Trials guidelines.

### Measures

In the home interviews, participants confirmed that they met eligibility criteria. They also described their cultural background, first language and housing situations. As well, a comprehensive array of validated scales and items were administered covering: additional health and social adversities (housing, mental health including prenatal substance use, physical health, history of maltreatment as a child, exposure to IPV in the past year); receipt of health and social services; maternal psychological resources (self-efficacy, mastery); and maternal cognitive ability and executive functioning. To enhance accuracy, field interviewers verbally administered questionnaires and cognitive tests in-person. Sensitive items deemed prone to reporting bias (such as prenatal substance use) were confidentially administered using headphones with audiotaped questions; participants then placed written responses in sealed envelopes for later processing by the study team. See Table 1.

**Table 1:** Summary of measures

Measurement Construct	Description	Scoring
Sociodemographic characteristics		
	Age, marital status, cultural background, first language, education, income and housing. Income was defined as pre-tax annual income from all sources of employment including unreported income and excluding any money received from family, friends or income assistance [28].	Descriptives.
Psychological resources		
Self-efficacy	General Self-Efficacy Scale [29]. Likert scale 10 items, e.g., “I am certain that I can accomplish my goals.”	Higher scores represent higher levels of self-efficacy.
Mastery	Pearlin Mastery Scale [30]. Likert scale 7 items, e.g., “I have little control over the things that happened to me.”	Higher scores represent higher levels of mastery.
Cognitive ability		
	Shipley-II [31]. Vocabulary Subscale 40 items assesses acquired knowledge. Abstraction Subscale 25 items assesses abstract reasoning.	Higher total raw scores indicate better performance.
Executive functioning		
Inhibition of interference	Stroop Colour and Word Test [32]. Cognitive assessment of ability to inhibit interference in the reaction time of a task.	Higher raw scores represent better cognitive performance.
Visual attention and task switching	Trail Making Test [33, 34]. Participants were timed while first sequentially connecting numbered circles (1–2, 2–3, etc.; TMT-A), then lettered and numbered circles (1-A, A-2, 2-B, etc.; TMT-B).	Shorter times represent better scores. A ratio of TMT-B / TMT-A represents executive control [35].
Socioeconomic disadvantage		
Having low income	Pre-tax annual income from all sources of employment including unreported income and excluding any money received from family, friends or income assistance.	Living on low income at <$20,000 annual employment income.
Having limited education	Not completing the equivalent of a BC high school diploma.	Dichotomous (yes/no) variable.
Being single (having limited social supports)	Not married or common-law (living together consecutively for one year or more).	Dichotomous (yes/no) variable.
Homelessness	Living on the streets or in an emergency or homeless shelter, staying in places not meant as residences, (e.g., car or tent), and/or experiencing “hidden homelessness”, (e.g., staying with someone because of no permanent address or having nowhere else to live or “couch-surfing”) [35, 36].	Dichotomous (yes/no) variable.
Unstable housing	Having to move three or more times or experiencing homelessness (past year).	Dichotomous (yes/no) variable.
History of child maltreatment		
Child maltreatment age 16 years or younger	Childhood Trauma Questionnaire – Short Form [37]. Likert scale 28 items, e.g., “When I was growing up, I didn’t have enough to eat.”	Moderate-to-severe levels of any type of abuse or neglect.
Exposure to intimate partner violence		
Including physical abuse, emotional abuse and harassment	Composite Abuse Scale [38]. Likert scale 30 items, e.g., “My partner told me that I wasn’t good enough.” Partner was defined as husband/wife, partner or boy/girlfriend for longer than one month.	Higher scores indicate higher levels of abuse.
Mental and physical health		
Psychological distress	Kessler Psychological Distress Scale [39]. Likert scale 10 items, e.g., “About how often did you feel hopeless?”	Total scores of > 25 represent moderate-to-severe anxiety or depression.
Mental and physical health conditions	Any long-term health conditions diagnosed by a physician affecting day-to-day activities [40, 41].	Number and type of diagnosed conditions.
Prenatal substance use		
Nicotine, alcohol, cannabis, and other street drugs	Frequency of use [42].	Dichotomous (yes/no) variables.
Receipt of health and social services		
Health services received for physical concerns	Visiting primary healthcare providers (family doctors, nurse, and midwives) and receiving prenatal classes.	Number and type of services.
Social services received	Income assistance through provincial or federal programs such as: BC Income and Disability Assistance, Canada Disability Benefits and Employment Insurance, BC Hardship Assistance, and BC Youth Agreements.	Number and type of services.

### Cumulative disadvantage

The proportion of participants experiencing between one and eight indicators of disadvantage was calculated. Indicators included: living on low income (less than $20,000 annually CAD); having limited education (less than high school); preparing to parent while single; experiencing unstable housing (having to move three or more times or experiencing homelessness in the past year); experiencing moderate/severe levels of psychological distress; having any prenatal substance use in the past month; having been maltreated as a child; and experiencing IPV within the past year.

### Statistical analyses

Descriptive statistics were used to characterize all variables. The data were screened for: accuracy of entry; patterns of missing data; and assumptions of normality, independence and homoscedasticity. To compare the two age groups (14–19 versus 20–24 years), we used the Chi-square test (or the Fisher’s exact test for cell sizes less than five). For continuous variables, we utilized the Student’s *t*-test. Statistical significance was set at *p*-value ≤0.05. In each table, *n* may be different from *N* due to missing data (for example, participants could choose not to respond to given items).

## Results

Data are provided on the total cohort as well as on the two age groups in Tables 2, 3, 4, 5 and 6. Missing data were minimal at less than 2% for all variables, except for unstable housing (3% missing) and English as a first language (7% missing). Central descriptives on the total cohort and statistically significant age group differences are summarized below.

**Table 2:** Baseline socioeconomic disadvantage according to eligibility criteria

		Age Group		*p*-value
	Total *N* = 739	14–19 years *N* = 361	20–24 years *N* = 378	
	*n* (%)	*n* (%)	*n* (%)	
Low income (living on < $20,000 annually)	606/726 (83.5)	319/354 (90.1)	287/372 (77.2)	**< 0.001**
Limited education (no high school completion)	384/738 (52.0)	246/360 (68.3)	138/378 (36.5)	**< 0.001**
Preparing to parent while single (not married or common-law)	670/736 (91.0)	312/360 (86.7)	358/376 (95.2)	**< 0.001**

Results in bold: *p*-value<0.05

**Table 3:** Sociodemographic characteristics, psychological resources and cognitive functioning

		Age Group		*p*-value
	Total *N* = 739	14–19 years *N* = 361	20–24 years *N* = 378	
	*n* (%)	*n* (%)	*n* (%)	
Sociodemographic Characteristics				
Cultural background^a^	*n* = 738	*n* = 361	*n* = 378	0.099
White	418 (56.6)	193 (53.5)	225 (59.5)	
Indigenous including First Nations, Métis and Inuit	79 (10.7)	36 (10.0)	43 (11.4)	
Indigenous including First Nations, Métis and Inuit and Other	121 (16.4)	73 (20.2)	48 (12.7)	
Mixed Heritage ≥2	55 (7.4)	30 (8.3)	25 (6.6)	
Asian (Chinese, S. Asian, or Other)	32 (4.3)	14 (3.9)	18 (4.8)	
Other (e.g., Latin-American, Black)	34 (4.6)	15 (4.2)	19 (5.0)	
English as first language	686 (93.0)	341 (94.7)	345 (91.3)	0.091
Highest educational qualification	*n* = 738	*n* = 360	*n* = 378	**< 0.001**
Less than high school	384 (52.0)	246 (68.3)	138 (36.5)	
High school or equivalent	270 (36.6)	103 (28.6)	167 (44.2)	
College or university degree	84 (11.4)	11 (3.1)	73 (19.3)	
Income from employment (annual CAD)	*n* = 726	*n* = 354	*n* = 372	**< 0.001**
Less than $5000	308 (42.4)	203 (57.3)	105 (28.2)	
$5000 – 9999	118 (16.3)	60 (16.9)	58 (15.6)	
$10,000 – 19,999	180 (24.8)	56 (15.8)	124 (33.3)	
$20,000 – 29,999	75 (10.3)	21 (5.9)	54 (14.5)	
$30,000 or more	45 (6.2)	14 (4.0)	31 (8.3)	
Current Housing	*n* = 725	*n* = 357	*n* = 368	0.138
House, apartment or condominium	681 (93.9)	332 (93.0)	349 (94.8)	
Group home, shelter or foster home	18 (2.5)	13 (3.6)	5 (1.4)	
Other (e.g., mobile home/trailer, single-room occupancy residence)	26 (3.6)	12 (3.4)	14 (3.8)	
	Mean (*SD*)	Mean (*SD*)	Mean (*SD*)	
Income from employment (annual CAD)	9928 (10575)	6811 (8976)	12,886 (11125)	**< 0.001**
Age	19.76 (2.36)	17.73 (1.17)	21.69 (1.40)	**< 0.001**
Psychological Resources				
Self-Efficacy	32.28 (3.93)	32.08 (3.79)	32.47 (4.05)	0.179
Mastery	21.42 (3.06)	21.53 (3.08)	21.32 (3.05)	0.361
Cognitive Functioning				
Shipley 2 – Vocabulary	24.35 (5.12)	23.53 (4.76)	25.13 (5.33)	**< 0.001**
Shipley 2 – Abstraction	11.89 (3.65)	11.73 (3.57)	12.04 (3.73)	0.248
Executive functioning				
Stroop Colour-Word Task Score	43.99 (9.19)	42.46 (8.64)	45.45 (9.46)	**< 0.001**
Stroop Interference Score	5.39 (6.72)	4.48 (6.15)	6.26 (7.12)	**< 0.001**
Trail Making Test B^b^	70.55 (32.67)	73.92 (33.23)	67.32 (31.84)	**0.006**
Trail Making Test B /A^b^	44.89 (29.28)	47.81 (29.87)	42.07 (28.45)	**0.008**

^a^Participants could give more than one answer; ^b^Shorter scores represent better performance on a timed task. Results in bold: *p*-value<0.05

**Table 4:** Health and social adversities including maltreatment experiences

		Age Group		*p*-value
	Total *N* = 739	14–19 years *N* = 361	20–24 years *N* = 378	
	*n* (%)	*n* (%)	*n* (%)	
Unstable housing				
Lifetime homelessness (including currently)	333/716 (47.0)	154/351 (44.0)	179/365 (49.0)	
Currently homeless	22/721 (3.1)	9/351 (2.6)	13/370 (3.5)	0.600
Moved ≥3 times or homeless (past year)	385/731 (52.1)	183/357 (51.3)	202/374 (54.0)	0.503
Psychological Distress (past month)				
Moderate/severe psychological distress	235/737 (31.9)	100/360 (27.8)	135/377 (35.8)	**0.024**
Mental health conditions^a^	*n* = 739	*n* = 361	*n* = 378	
Severe anxiety or depression regularly	346 (46.8)	173 (47.9)	173 (45.8)	0.608
Diagnosed mental disorder (e.g., bipolar disorder or attention problems)	160 (21.7)	73 (20.2)	87 (23.0)	0.405
Diagnosed developmental conditions (e.g., autism spectrum or learning disorders)	83 (11.2)	35 (9.7)	48 (12.7)	0.240
Prenatal substance use				
Any cannabis, alcohol or street drug use (past month)^a^	172/732 (23.5)	80/357 (22.4)	92/375 (24.5)	0.555
Cannabis use (past month)	155/738 (21.0)	75/360 (20.8)	80/378 (21.2)	0.984
Alcohol use (past month)	17/736 (2.3)	6/361 (1.7)	11/375 (2.9)	0.367
Street drug use (past month)	11/736 (1.5)	< 5/358 (< 2)	7/378 (1.9)	0.605
Nicotine/cigarette use (past 48 h)	196/736 (26.6)	96/360 (26.7)	100/376 (26.6)	> 0.999
Second-hand smoke exposure (past week)	292/736 (39.7)	150/361(41.6)	142/375 (37.9)	0.344
Serious long-term physical health conditions^a^ *n* = 739		*n* = 361	*n* = 378	
Iron-deficiency anemia	151 (20.4)	69 (19.1)	82 (21.7)	0.437
Asthma or allergies (regular use of puffers)	139 (18.8)	64 (17.7)	75 (19.8)	0.522
Migraines (weekly or more)	108 (14.6)	57 (15.8)	51 (13.5)	0.436
Serious injury (head/leg) that left a disability	57 (7.7)	24 (6.6)	33 (8.7)	0.356
Thyroid disease	21 (2.8)	6 (1.7)	15 (4.0)	0.096
Cardiovascular disease (including high blood pressure)	13 (1.8)	< 5 (< 2)	9 (2.4)	0.300
Epilepsy or seizures (weekly or more)	13 (1.8)	7 (1.9)	6 (1.6)	0.933
Other (e.g., arthritis, irritable bowel syndrome, autoimmune disorders)	126 (17.1)	61 (16.9)	65 (17.2)	0.992
Maltreatment experiences^a^				
Maltreatment at age 16 years or younger				
Moderate/severe neglect, physical abuse, emotional abuse and/or sexual abuse	410/728 (56.3)	196/355 (55.2)	214/373 (57.4)	0.608
Intimate partner violence in past year				
Any physical abuse, emotional abuse and harassment	363/734 (49.5)	181/358 (50.6)	182/376 (48.4)	0.61

^a^Participants could give more than one answer. Results in bold: *p*-value<0.05

**Table 5:** Receiving health and social services^a^

		Age Group		*p*-value
	Total *N* = 739	14–19 years *N* = 361	20–24 years *N* = 378	
	*n* (%)	*n* (%)	*n* (%)	
Health services for physical health				
Primary healthcare (past month)	567/739 (76.7)	289/361 (80.1)	278/378 (73.5)	**0.045**
Prenatal classes (past month)	210/739 (28.4)	116/361 (32.1)	94/378 (24.9)	**0.035**
Social services received				
Income assistance (past month)	212/739 (28.7)	71/361 (19.7)	141/378 (37.3)	**< 0.001**

^a^Participants could give more than one answer. Results in bold: *p*-value<0.05

**Table 6:** Cumulative disadvantage

	Total *N* = 739	Age Group	
		14–19 years *N* = 361	20–24 years *N* = 378
Indicators of disadvantage	*n* (%)	*n* (%)	*n* (%)
1	26 (3.5)	12 (3.3)	14 (3.7)
2	73 (9.9)	27 (7.5)	46 (12.2)
3	119 (16.1)	61 (16.9)	58 (15.3)
4	140 (18.9)	67 (18.6)	73 (19.3)
5	152 (20.6)	68 (18.8)	84 (22.2)
6	121 (16.4)	68 (18.8)	53 (14.0)
7	77 (10.4)	35 (9.7)	42 (11.1)
8	29 (3.9)	21 (5.8)	8 (2.1)

### Baseline socioeconomic disadvantage according to eligibility criteria

Nearly half of participants (49%) were aged 14–19 years, while just over half (51%) were aged 20–24 years. Most of the cohort (84%) were preparing to parent while living on low income (less than $20,000 CAD annually); more than half (52%) had not completed high school (including 182 pregnant girls or 25% of the total cohort who were still attending high school); and almost all (91%) were preparing to parent while single. Compared to young women, more girls reported living on low income and having limited education, but fewer were preparing to parent while single. See Table 2.

### Sociodemographic characteristics

Most participants (57%) identified as “white”, while over a quarter (27%) identified as Indigenous (including First Nations, Métis or Inuit) and others identified as mixed heritage (7%), Asian (4%) or other cultural backgrounds (5%). Most (93%) reported English as their first language (in keeping with eligibility criteria requiring conversational competence in English). See Table 3.

### Psychological resources and cognitive functioning

The mean raw scores for self-efficacy, mastery, cognitive ability and executive functioning are presented in Table 3. Compared to girls, young women had significantly better performance on measures of cognitive ability (vocabulary) and executive functioning.

### Health and social adversities including maltreatment experiences

Participants experienced health and social adversities beyond those associated with the eligibility criteria including: lifetime homelessness (47%); housing instability (52%); moderate/severe psychological distress (32%); severe anxiety or depression (47%); other diagnosed mental disorders (22%); prenatal nicotine and cannabis use (27 and 21% respectively); physical health problems (20%); child maltreatment when younger (56%); and exposure to IPV recently (50%). More young women (36%) also reported moderate/severe psychological distress compared to girls (28%). See Table 4.

### Receiving health and social services

Most participants (77%) reported visiting primary healthcare providers (physicians, nurse practitioners and/or midwives) regarding physical health concerns in the past month. Less than a third (28%) received prenatal classes. As well, despite most (84%) living on low income, less than a third (29%) reported receiving social benefits such as income assistance or other BC or Canadian entitlements. More girls compared to young women received primary healthcare (80% versus 74%) and prenatal classes (32% versus 25%) in the past month; whereas, more young women (37%) compared to girls (20%) received income assistance. See Table 5.

### Cumulative disadvantage

Almost all participants (96%) were experiencing two or more indicators of adversity. As well, more than two thirds (70%) were experiencing four or more indicators. See Table 6.

## Discussion

Our data have depicted a cohort of pregnant girls and young women in BC, Canada, who were recruited to a trial based on selected indicators of socioeconomic disadvantage (young age, low income, limited education and/or single parenting). Yet the data indicated that all participants selected using these socioeconomic and demographic indicators were also experiencing substantial *additional* health and social adversities. These added adversities included: housing instability, mental and physical health problems including prenatal substance use, maltreatment during childhood, and IPV exposure recently. As well, despite BC’s existing social services, less than a third had received recent income assistance entitlements. Perhaps most telling, almost all were experiencing two or more indicators of adversity while more than two thirds were experiencing four or more — suggesting considerable cumulative disadvantage.

How does this cohort compare to other British Columbians and Canadians? Beyond the eligibility criteria, while directly comparable data were not available for all variables, our cohort nevertheless reported much higher rates of associated health and social adversities including: homelessness and unstable housing, mental health problems including prenatal substance use, and serious physical health problems [43–50]. Rates of child maltreatment and IPV exposure were also twice those found for other Canadians [51, 52]. These comparisons confirm that we recruited a cohort who was experiencing marked disadvantage, the population NFP is most intended to benefit. See Table 7.

**Table 7:** Indicators of adversity in BCHCP cohort compared to other Canadians

	BCHCP population		Canadian samples (%)	
	14–19 years (%)	20–24 years (%)	%	
BCHCP screening criteria				
Young age during pregnancy	49	51	2–11	Canadian females ≤19 years (2%) and 20–24 years (11%) [43]
Low income (< $20,000 per year)	90	77	13–15	Canadian females < 18 years (15%) and ≥ 18 years (13%) [44]
Limited education (no high school completion)	69	37	17	BC females of all ages [45]
Preparing to parent while single (not married or common-law)	87	95	7–45	Canadian females ≥15 years (7%) and living on low income (45%) [46]
Additional health and social adversities				
Homeless ever (including currently)	44	49	5	Canadians (male and female) 15–24 years [47]
Unstable housing (moving in past year)	35	33	20	Canadian females ≥15–24 years [47]
Psychological distress	28	36	30	Canadian females ≥15 years and low-income [48]
Diagnosed mental disorder	20	23	15	Canadian females ≥15 years [48]
Prenatal cannabis use	21	21	15	BC females 12–17 years [49]
Prenatal nicotine use	27	27	22	Canadian females ≥15 years and low income [50]
Serious physical health problems	≤ 19	≤ 22	13	BC females 12–17 years [49]
Child maltreatment when ≤16 years	55	57	30	Canadian females ≥15 years [51]
Exposure to intimate partner violence in recent past	51	48	22–40	Canadian females 15–19 years (40%) and 20–24 years (22%) [52]

What have our data added? We have shown for the first time that high levels of disadvantage exist for some young Canadians — despite this country’s high-income status and its longstanding commitment to equity in access to universal healthcare and related social services [54]. As well, despite provincial/territorial variations in the delivery of health and social programs [53], our data nevertheless have national implications. In Canada, approximately 8000 children are born to adolescent mothers each year [43], while approximately 42,000 are born to young mothers (aged 20–24 years), with many of the latter experiencing low income (13%) and/or single parenthood (7%) [43, 46]. Reaching these populations and addressing avoidable adversities during early pregnancy — thereby also increasing children’s life chances — is a societal imperative [16, 18, 20]. Our data also suggest that public policy remedies must extend beyond public health and healthcare — encompassing social services, such as ensuring adequate housing and incomes, and preventing child maltreatment and IPV as early as possible in the lifespan. We believe that our study therefore provides new data underscoring an urgent call to action across public sectors not only in BC, but also in Canada.

Our data also have implications for children’s rights. We found less than a third of participants had recently received social service entitlements such as income assistance, while approximately half reported recent unstable housing and lifetime homelessness as well as exposure to child maltreatment and IPV. Addressing these serious avoidable adversities is a priority, especially for pregnant youth [1, 4, 7, 11, 54]. According to international child rights’ conventions, BC and other provinces/territories also have obligations to ensure that the basic needs of all minors are met, including protecting young people from harm and ensuring adequate housing, income and parental/caregiver supports [55, 56]. Our data suggest that these fundamental obligations may not be being fulfilled in BC.

Regarding age differences, we found that in this cohort, young women were facing adversities that were comparable to girls. The statistically significant differences that we did observe between the two groups may be explained by developmental stage (differences in cognitive functioning), eligibility criteria (being single was only a criteria for young women), or developmental context (lower reported income and education may be expected for the pregnant girls who may still be in school and less likely to be employed). The higher proportion of girls accessing primary healthcare and prenatal classes may be a result of better provision of services for these pregnant adolescents compared with young women, although further data are needed. Yet overall, our data suggest that the well-established risks facing children born to adolescents may also extend to children of young women who are experiencing socioeconomic disadvantage in BC.

The BCHCP RCT is embedded within BC’s health system, with NFP being delivered as an enhanced public health service — an example of delivering services proportionate to need [18]. Participants will be followed throughout pregnancy and until their children reach age 2 years (the duration of the NFP program). Additional outcome findings will be available in 2020–2022. The embedded nature of this RCT ensures that findings are shared quickly and efficiently with policy and practice partners to inform ongoing strategies to better reach populations in need.

There are nevertheless limitations to the data reported here. This cohort was not a representative sample nor did it represent all the potentially eligible girls and young women, in that many (two thirds) were not reached through BCHCP recruitment efforts. Further collaborative research-practice-policy efforts are needed to better identify and provide services and supports for this “need-to-reach” population. We also acknowledge that the data on education levels does not account for those girls who were still attending high-school (*n* = 182 or 25% of the total cohort). However, all girls were pregnant and preparing to parent at a young age such that their education and employment opportunities were interrupted, placing them and their children at risk for disadvantage.

## Conclusions

Our data suggest that unacceptably high levels of socioeconomic disadvantage exist for some young British Columbians — despite existing health and social services in a high-income province in a high-income country. Concentrated disadvantage for mothers also places children at risk for a range of adversities and for long-term developmental and mental health problems. Therefore, greater health and social supports and services are warranted for this population — to help them and to help their children.

## Data Availability

All data supporting the results are included in this article.

## Abbreviations

BC: British Columbia
BCHCP: British Columbia Healthy Connections Project
CAD: Canadian dollar
IPV: Intimate partner violence
NFP: Nurse-Family Partnership
RCT: Randomized controlled trial
REB: Research Ethics Board

## References

[1] Aizer A, Currie J (2014). The intergenerational transmission of inequality: maternal disadvantage and health at birth. Science.

[2] Evans G (2016). Childhood poverty and adult psychological well-being. Proc Natl Acad Sci U S A.

[3] Reiss F (2013). Socioeconomic inequalities and mental health problems in children and adolescents: a systematic review. Soc Sci Med.

[4] Luong M (2008). Life after teenage motherhood. Perspectives on labour and income.

[5] Chico E, Gonzalez A, Ali N, Steiner M, Fleming AS (2014). Executive function and mothering: challenges faced by teenage mothers. Dev Psychobiol.

[6] Ekéus C, Christensson K, Hjern A (2004). Unintentional and violent injuries among pre-school children of teenage mothers in Sweden: a national cohort study. J Epidemiol Community Health.

[7] Jutte DP, Roos NP, Brownell MD, Briggs G, MacWilliam L, Roos LL (2010). The ripples of adolescent motherhood: social, educational, and medical outcomes for children of teen and prior teen mothers. Acad Pediatr.

[8] Putnam-Hornstein E, Cederbaum JA, King B, Eastman AL, Trickett PK (2015). A population-level and longitudinal study of adolescent mothers and intergenerational maltreatment. Am J Epidemiol.

[9] Gilbride SJ, Wild C, Wilson DR, Svenson LW, Spady DW (2006). Socio-economic status and types of childhood injury in Alberta: a population based study. BMC Pediatr.

[10] Lefebvre R, Fallon B, Van Wert M, Filippelli J (2017). Behav Sci.

[11] Oh DL, Jerman P, Silvério Marques S, Koita K, Purewal Boparai SK, Burke Harris N (2018). Systematic review of pediatric health outcomes associated with childhood adversity. BMC Pediatr.

[12] Orton E, Kendrick D, West J, Tata LJ (2012). Independent risk factors for injury in pre-school children: three population-based nested case-control studies using routine primary care data. PLoS One.

[13] Goodman SH, Rouse MH, Connell AM, Broth MR, Hall CM, Heyward D (2011). Maternal depression and child psychopathology: a meta-analytic review. Clin Child Fam Psychol Rev.

[14] Grant KS, Petroff R, Isoherranen N, Stella N, Burbacher TM (2018). Cannabis use during pregnancy: pharmacokinetics and effects on child development. Pharmacol Ther.

[15] Huang C-C, Vikse JH, Lu S, Yi S (2015). Children’s exposure to intimate partner violence and early delinquency. J Fam Violence.

[16] World Health Organization and Calouste Gulbenkian Foundation (2014). Social determinants of mental health.

[17] Braveman P, Egerter S, Williams DR (2011). The social determinants of health: coming of age. Annu Rev Public Health.

[18] Marmot M, Allen J, Goldblatt P, Boyce T, McNeish D, Grady M (2010). Fair society, healthy lives.

[19] Heckman JJ (2006). Skill formation and the economics of investing in disadvantaged children. Science.

[20] Waddell C, Schwartz C, Andres C (2018). Making children’s mental health a public policy priority: for the one and the many. Public Health Ethics.

[21] MacMillan HL, Wathen CN, Barlow J, Fergusson DM, Leventhal JM, Taussig HN (2009). Interventions to prevent child maltreatment and associated impairment. Lancet.

[22] Hertzman C, Boyce T (2010). How experience gets under the skin to create gradients in developmental health. Annu Rev Public Health.

[23] Olds DL (2008). Preventing child maltreatment and crime with prenatal and infancy support of parents: the nurse-family partnership. J Scand Stud Criminol Crime Prev.

[24] Bonevski B, Randell M, Paul C, Chapman K, Twyman L, Bryant J (2014). Reaching the hard-to-reach: a systematic review of strategies for improving health and medical research with socially disadvantaged groups. BMC Med Res Methodol.

[25] Catherine NLA, Gonzalez A, Boyle M, Sheehan D, Jack SM, Hougham KA (2016). Improving children’s health and development in British Columbia through nurse home visiting: a randomized controlled trial protocol. BMC Health Serv Res.

[26] Robling M, Bekkers MJ, Bell K, Butler CC, Cannings-John R, Channon S (2016). Effectiveness of a nurse-led intensive home-visitation programme for first-time teenage mothers (building blocks): a pragmatic randomised controlled trial. Lancet.

[27] Mejdoubi J, van den Heijkant SC, van Leerdam FJ, Crone M, Crijnen A, HiraSing RA (2014). Effects of nurse home visitation on cigarette smoking, pregnancy outcomes and breastfeeding: a randomized controlled trial. Midwifery.

[28] Statistics Canada (2010). 2006 Census of Population.

[29] Schwarzer R, Jerusalem M, Weinman J, Wright S, Johnston M (1995). Generalized self-efficacy scale. Measures in health psychology: a user’s portfolio.

[30] Pearlin LI, Schooler C (1978). The structure of coping: erratum. J Health Soc Behav.

[31] Shipley W, Gruber C, Martin T, Klein A (2009). Shipley-2.

[32] Golden C (1978). Stroop color and word test: cat. No. 30150M. In: a manual for clinical and experimental uses.

[33] Reitan R, Wolfson D (1993). The Halstead-Reitan Neurospychological test battery.

[34] Sánchez-Cubillo I, Periáñez JA, Adrover-Roig D, Rodríguez-Sánchez JM, Ríos-Lago M, Tirapu J (2009). Construct validity of the trail making test: role of task-switching, working memory, inhibition/interference control, and visuomotor abilities. J Int Neuropsychol Soc.

[35] Gaetz S, Barr C, Friesen A, Harris B, Hill C, Kovacs-Burns K (2012). Canadian definition of homelessness.

[36] Greater Vancouver Regional Steering Committee on Homelessness (2014). Results of the 2014 homeless count in the Metro Vancouver Region.

[37] Bernstein DP, Stein JA, Newcomb MD, Walker E, Pogge D, Ahluvalia T (2003). Development and validation of a brief screening version of the childhood trauma questionnaire. Child Abuse Negl.

[38] Hegarty K, Bush R, Sheeham M (2005). The composite abuse scale: further development and assessment of reliability and validity of a multidimensional partner abuse measure in clinical settings. Violence Vict.

[39] Kessler RC, Andrews G, Colpe LJ, Hiripi E, Mroczek DK, Normand S-LT (2002). Short screening scales to monitor population prevalences and trends in non-specific psychological distress. Psychol Med.

[40] Statistics Canada (2011). Canadian community health survey annual component.

[41] Statistics Canada (2012). Canadian health measures survey (Cycle 2).

[42] Statistics Canada (2009). National Longitudinal Survey of Children and Youth (Cycle 8).

[43] Statistics Canada (2018). Live births, by age of mother.

[44] Statistics Canada (2019). Low income statistics by age, sex and economic family type, Table 11-10-0135-01.

[45] Statistics Canada (2017). Education indicators in Canada: An international perspective.

[46] Urquia ML, O’Campo PJ, Ray JG (2013). Marital status, duration of cohabitation, and psychosocial well-being among childbearing women: a Canadian nationwide survey. Am J Public Health.

[47] Rodrigue S (2016). Hidden homelessness in Canada.

[48] Caron J, Liu A (2010). A descriptive study of the prevalence of psychological distress and mental disorders in the Canadian population: comparison between low-income and non–low-income populations. Chronic Dis Can.

[49] Smith A, Saewyc E, Forsyth K, Poon C, Peled M, Martin S (2019). Balance and connection in BC: the health and well-being of our youth. Results of the 2018 BC adolescent health survey.

[50] Al-Sahab B, Saqib M, Hauser G, Tamim H (2010). Prevalence of smoking during pregnancy and associated risk factors among Canadian women: a national survey. BMC Pregnancy Childbirth.

[51] MacMillan HL, Boyle M, Taillieu T, Cheung K, Sareen J, Afifi TO (2014). Child abuse and mental disorders in Canada. CMAJ.

[52] Daoud N, Urquia ML, O’Campo P, Heaman M, Janssen PA, Smylie J (2012). Prevalence of abuse and violence before, during, and after pregnancy in a national sample of Canadian women. Am J Public Health.

[53] Waddell C, Georgiades K, Duncan L, Comeau J, Reid GJ, O’Briain W (2019). The 2014 Ontario child health study findings: policy implications for Canada. Can J Psychiatr.

[54] Allen J, Balfour R, Bell R, Marmot M (2014). Social determinants of mental health. Int Rev Psychiatry.

[55] British Columbia Government (1996). Child, Family and Community Service Act.

[56] United Nations (1989). Convention on the Rights of the Child.

